# Machining Micro-Error Compensation Methods for External Turning Tool Wear of CNC Machines

**DOI:** 10.3390/mi16101143

**Published:** 2025-10-08

**Authors:** Hui Zhang, Tongwei Lu, Zhijie Xia, Zhisheng Zhang, Jianxiong Zhu

**Affiliations:** 1 School of Mechanical and Electronic Engineering, Nanjing Forestry University, Nanjing 210037, China; 2 Hubei Key Laboratory of Intelligent Robot, Wuhan Institute of Technology, Wuhan 430205, China; 3 School of Mechanical Engineering, Southeast University, Nanjing 211189, China

**Keywords:** linear detection, visual model, external turning tool, tool nose wear, error compensation

## Abstract

Tool wear detection is very important in CNC machine tool cutting. Once the tool is excessively worn, it is not only easy to cause the workpiece to be scrapped, but even to damage the machine. Therefore, common external turning tools of CNC machines are studied. The effect of tool nose wear on machining accuracy was analyzed by a building mathematical model. According to different wear conditions, a linear detection method based on edge images and input features was proposed to detect the main and secondary cutting edges, which helped determine the theoretical center of the tool nose and build a morphological visual model. For different error cases, the axial and radial error compensation strategies were proposed, respectively. By comparing the experimental data of four kinds of workpieces before and after compensation machining, the average errors of them were reduced separately, and the maximum value reached 79.2%, which verified the effectiveness of the compensation strategy. The intelligent compensation strategies will significantly improve the micro-machining accuracy and efficiency of the external turning tools in CNC machines.

## 1. Introduction

In the CNC turning process, the tool nose arc is easy to wear, so that the actual radius of the tool nose is less than the theoretical value, which affects the machining accuracy of workpieces [1]. Measuring the actual radius of the tool nose is helpful to compensate for the machining error [2]. Timely grasp of tool nose wear is beneficial to improve the workpiece processing quality and production efficiency, and reduce the occurrences of accidents [3,4].

The core idea of the compensation is to neutralize or greatly weaken the existing error. By mastering the characteristics and rules of the machining error, the mathematical model has been built to improve the dimensional machining accuracy of the tools. Traditional error compensation mainly relies on hardware implementation, which has low efficiency and a lack of flexibility. The method of software detection and compensation is more conducive to efficiently improving the manufacturing accuracy [5,6,7].

There are two main methods for tool wear detection: direct and indirect measurements. The indirect measurement method can infer the tool wear through cutting process signals obtained by various sensors. However, this kind of method cannot accurately measure the wear values [8,9,10]. Direct measurement method obtains the wear value by measuring the workpiece or tool size changes under complex lighting environments [11]. The accurate position and the wear level are difficult to obtain in the tool wear region [12]. Therefore, machine vision as a direct measurement method, with the advantages of non-contact and accurate measurement, has become an important means to detect the tool wear states [13].

Conventional tool condition monitoring also cannot meet the high precision requirement in micro machining. Therefore, multi-scale dictionaries instead of the large-scale training for super-resolution reconstruction were developed [14]. Machine learning extracts representations from the images, which helps machines to learn autonomously and understand the environment [15].

In the image processing field, line detection is a basic and important task. This technology provides a reliable image recognition and analysis method, which is for application scenarios, by accurately detecting the line or line segments [16]. It mainly includes three methods: the Hough transform, image edge detection, and the image gradient method [17,18]. The Hough transform method is beneficial to lower complexity and save time [19]. The image gradient method has high detection accuracy and stability [20]. Rasiq and Krishnakumar [21] proposed an object recognition method that extracted object features using high-speed color segmentation and fast straight-line extraction. Li et al. [22] introduced the Non-Uniform Rational B-Spline (NURBS) tool radius compensation algorithm, which could directly deal with the contour trajectory, and output the tool center trajectory in straight-line interpolation G code. However, if the contour trajectory contained too many NURBS, the calculation speed of the algorithm would be slow.

CNC machining compensation methods can be divided into online and offline compensation. Offline compensation is a strategy adopted for new processing based on the error compensation model, which is applied in the machining gap. Online compensation is based on the real-time processing of information. Maeng and Min [23] utilized an online measurement system to identify both the geometric error of the rotation axis and tool alignment errors. Li et al. [24] presented a precision degradation modeling approach based on a meta-action unit for CNC machine tools, and the motion transmission ratio was used to synthesize multiple error sources. Pu et al. [25] developed a R-Test device, and various geometric errors of the rotation axis were effectively identified.

The offline compensation method has higher efficiency and lower cost. Offline modification of machining codes is more suitable for the turning process, which is used to compensate for the error caused by the tool nose wear on the front tool surface. Li et al. [26] proposed an identification method for the geometric error of a five-axis machine tool. And a laser tracker was used to collect error data in a large range. The volumetric error of any position in a workspace could be predicted and offline-compensated.

In this study, a tool wear error compensation system based on machine vision is established, and a compensation strategy is designed. The wear area of the normal wear tool was completely extracted, and the wear value was measured to determine whether the tool met the blunt standard. The tool that met the blunt standard would be discarded. If the tools did not reach the blunting standard, the tool nose radius wear values of the front tool surface were further measured. Then the machining codes were modified to compensate. Cutting path images were generated on the Web platform to ensure the accuracy and stability of the machining process.

## 2. Design of CNC External Turning Tool Wear Detection

**Figure 1:**
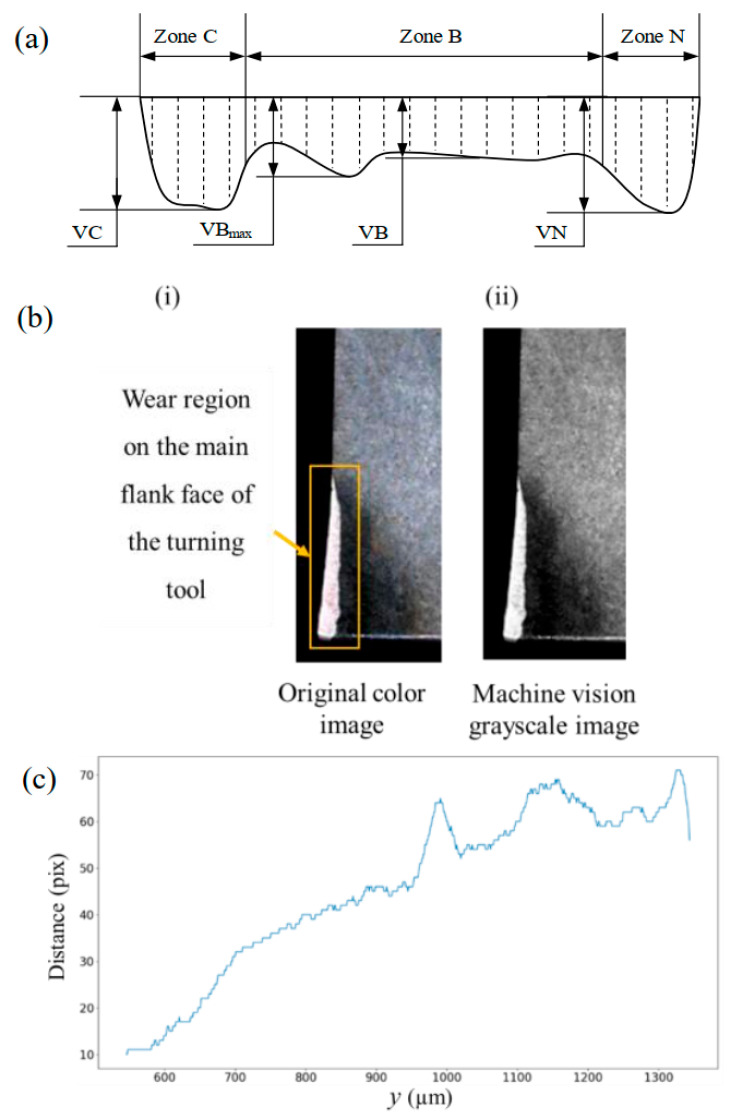
(**a**) The schematic of flank face wear on cutting tools. (**b**) The original (**i**) and grayscale image (**ii**) of turning tool wear. (**c**) Pixel distance (pix) of the wear value in *y*-axis direction (µm) in zone B.

The primary flank face of an external turning tool is in contact with the workpiece surface, where the wear phenomena are common, and elastoplastic deformation occurs due to the high pressure. The wear region is usually composed of a C zone, N zone, and B zone with irregular characteristics. Wear zone B is more uniform, with its average width represented by *VB* and *VB_max_* representing the maximum width in the *y*-axis direction. The N zone is often called the boundary wear, with *VN* representing its maximum width. The C zone is close to the tool nose, and the heat dissipation is not good, so the wear value is large. Its maximum wear width is represented by *VC* as shown in Figure 1a. The normal wear image of the main flank face collected in the experiment is shown in Figure 1b(i). The rectangular box represents the turning tool wear region, which indicates that the width of the wear region is relatively uniform. Its image obtained using machine vision is shown in Figure 1b(ii). In order to measure the flank face wear value, five steps should be carried out successively: morphological processing, edge detection, curve fitting, wear value calculation, and blunt judgment. For some stains that cannot be accurately excluded, the extraction results of wear regions need to be optimized by morphological algorithms. The edge detection employs the Canny algorithm to extract the wear region edge.

Then the curve S can be obtained by fitting the left active cutting-edge curve. The pixel distance *D_i_* (pix), from the edge point in the wear area to the curve S in an image, is measured. Then the average pixel distance of *VB* and the maximum pixel distance of *VB_max_* are obtained. The actual distance is further gained by combining the pixel equivalent of the camera calibration. Then the *VB* value of the uniform wear zone and the *VB_max_* value of the non-uniform wear zone are judged. The pixel distance curve of uniform wear values *VB* is displayed in Figure 1c.

According to the international wear assessment standard, there are two kinds of blunting standards of CNC external turning tools. For the uniform wear zone, the dulling standard is that the wear value *VB* of the main back tool face does not exceed 0.3 mm. In non-uniform wear belts, the maximum wear width does not exceed 0.6 mm. The measurement results of the wear values are shown in Table 1, which do not reach the blunting standard, and need to further complete processing error compensation. Then a line segment detector (LSD) algorithm, based on edge images and input features, is proposed to detect the straight line and measure the tool nose radius. Visual modeling of the arc shape on the front tool surface is carried out to obtain the radius of the CNC external turning tool after wear. And the model is used for compensation after 3D part identification. CNC machining codes are further modified.

### 2.1. Shape Visual Modeling of the Tool Nose Rake Face

Two telecentric lenses produced by Hikvision, MVL-HT-07-110 and MVL-HT-1-110, were chosen in this work. An optical camera with a CMOS chip was used to acquire external turning tool images, and its specific model was MV-CE060-10UC. A backside light source that could highlight the contour of the cutting tool was selected. For the main flank face, a ring-shaped light source was adopted to enhance the contrast through multi-angle illumination.

Then Sobel operator was utilized to calculate the x and y components of the pixel gradient in external turning tool images. Then the gradient amplitude and each pixel direction were calculated in denoised images. Sobel templates for detecting the *x* and *y* directions are as follows:

(1) $$S_{x}=\left[\begin{array}{ccc} -1\; & 0 & \;1\\ -2\; & 0 & \;2\\ -1\; & 0 & \;1 \end{array}\right]$$

(2) $$S_{y}=\left[\begin{array}{ccc} 1 & 2 & 1\\ 0 & 0 & 0\\ -1 & -2 & -1 \end{array}\right]$$

The image pixel gradient amplitude is $M=\sqrt{G_{x}^{2}\left(x,y\right)+G_{y}^{2}\left(x,y\right)}$, and the angle is $\theta =arctan\left(G_{y}/G_{x}\right)$. $G_{x}$ and $G_{y}$ represent gradient components in the *x* and *y* directions calculated by the Sobel operator, respectively. The global gradient only reflects the edge intensity of the whole image; it cannot accurately determine the edge position. Therefore, gradient non-maximum suppression is required. Local maximum points are found in the gradient image, and all pixels except these points are suppressed to zero to accurately locate the image edge.

To further process the edge after non-maximum suppression, double-threshold detection is a key step in the Canny edge detection algorithm. For each weak edge point, check whether there is a point with a gradient amplitude greater than the strong threshold in its 8 neighborhoods. If there is at least one strong edge point, the weak edge point is marked as an edge and set to 255. If it does not exist, discard it and set it to 0. Through these two steps, double-threshold detection can mark the points with large gradient amplitudes as the strong edge. Connect some points with small gradient amplitudes, and these points still have certain edge characteristics to obtain the final edge image. Then the tool edge is fine and completely identified through Canny operator detection.

To eliminate or weaken the influence of sawtooth effect on the algorithm, the input image source is reduced to 80% of the original image. Then it is processed by Gaussian down sampling. LSD linear detection is used for grayscale images, and the gradient calculation is completed by using a 2 × 2 template, as shown in Equations (3) and (4). This small template is faster to calculate and is independent of the neighborhood distribution.

(3) $$G_{x}\left(x,y\right)=\frac{i\left(x+1,y\right)+i\left(x+1,y+1\right)-i\left(x,y\right)-i(x,y+1)}{2}$$

(4) $$G_{y}\left(x,y\right)=\frac{i\left(x,y+1\right)+i\left(x+1,y+1\right)-i\left(x,y\right)-i(x+1,y)}{2}$$

where $i\left(x,y\right)$ is the image gray value at the pixel $\left(x,y\right)$. The calculated $G_{x}\left(x,y\right)$ and $G_{y}\left(x,y\right)$ are component gradients of the pixels $\left(x+0.5,y+0.5\right)$.

In the LSD algorithm, a sharp change in the gradient amplitude indicates that there is a strong edge in the image. The center pixel usually has the maximum gradient amplitude. To effectively carry out linear detection, it is necessary to sort the gradient amplitudes of pixels, which identifies the area with the most significant gradient change. When calculating the pixel gradient in flat regions, the gradient calculation error increases due to the lack of obvious gray change. Therefore, the pixels whose gradient amplitudes are lower than the presetting threshold are excluded in LSD algorithm. The gradient threshold is calculated as $\rho =\frac{q}{\sin\tau }$, where the angle tolerance is *τ* = 22.5° and the upper limit of error is *q* = 2.

In the process of generating the linear support region, an initial support region is first set, and it only contains a pixel with the largest gradient value. The gradient direction of this pixel is established as the dominant direction of the support region, and it is marked as being used. The eight neighbor regions of this pixel are tested in detail. The pixels are incorporated into the linear support region, whose angle with the current region direction is less than the preset threshold *τ*. Meanwhile, these newly added pixels are marked. Recalculate the orientation of the line support region based on these pixels and ensure that the orientation is consistent with the gradient direction of all pixels. This process is repeated till no new pixels can be added to the region.

When the area contains all pixels that form a line, the direction is recalculated as $\varphi =\arctan\left(\frac{\sum_{i}\sin{(\theta }_{i})}{\sum_{i}\cos{(\theta }_{i})}\right)$. The direction of the line support region after each iteration is *φ*. Each pixel is traversed and identified by the index *i*. The rectangular fitting process of the region involves the calculation of each minimal external rectangle. The LSD line detection algorithm is based on the Helmholtz principle to verify the line. It defines the number of false alarms (NFA) in the line region and eliminates error lines that may exist in the initial detection. The linear support region is determined by the width and two ends of the main axis.

In an image with a resolution of *M* × *N*, the number of possible linear support regions is ${\left(NM\right)}^{2/5}$. The total number of test rectangles is ${\left(NM\right)}^{2/5}\times \gamma$, where γ is the controllable parameter, and it is set as γ=2. The total number of pixels is *n*. The number of pixels is *k* in the same direction as the fitted rectangle. For measuring the effectiveness of the linear support region, the precision parameter *p* is introduced, and the calculation equation of the *NFA* is as follows:

(5) $$NFA={\left(NM\right)}^{2/5}\times \gamma \times B\left(n,\;k,\;p\right)$$

where the binomial model $B\left(n,\;k,\;p\right)$ is given:

(6) $$B(n,\;k,\;p)=\sum_{j=k}^{n}\left(\begin{array}{c} n\\ j \end{array}\right)p^{j}\times {\left(1-p\right)}^{n-j}$$

By bringing Equation (6) into Equation (5), the following is obtained:

(7) $$NFA={\left(NM\right)}^{2/5}\times \gamma \times \sum_{j=k}^{n}\left(\begin{array}{c} n\\ j \end{array}\right)p^{j}{\left(1-p\right)}^{n-j}$$

**Figure 2:**
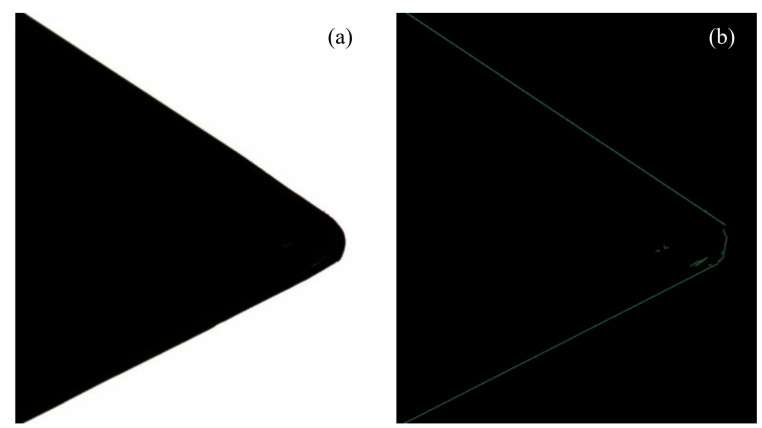
Detection results of the rake face image using LSD algorithm. (**a**) Original image. (**b**) LSD linear detection result.

If the *NFA* in the line support region is below a preset threshold, a line is detected. The results are obtained by using the LSD algorithm in the original rake face image as shown in Figure 2. The algorithm can initially detect the straight lines of the main and the secondary cutting edges, but the internal shape and stains of the rake face will interfere with the detection results of straight lines. The tool nose arc will also be detected as a multi-segment straight line, so that it is necessary to further improve the algorithm [27].

### 2.2. Tool Nose Straight Line Detection and Radius Measurement Algorithm Based on Edge Images and Input Features

Edge results are taken as the input data in the linear detection algorithm of the Canny operator. The Canny edge detection algorithm has characteristics of high precision and reduced noise, which can locate edges accurately. The LSD linear detection algorithm may miss some subtle edges when filtering pixels using the threshold. And the process involves several pixel gradient comparisons, which results in low efficiency. For further optimization, the Canny algorithm is used to extract the main and secondary cutting edges and the tool nose arc in a rake face image. Then the linear support region is searched on the rake face edge. This strategy helps improve the efficiency and robustness of the algorithm and can detect the linear features more accurately.

**Figure 3:**
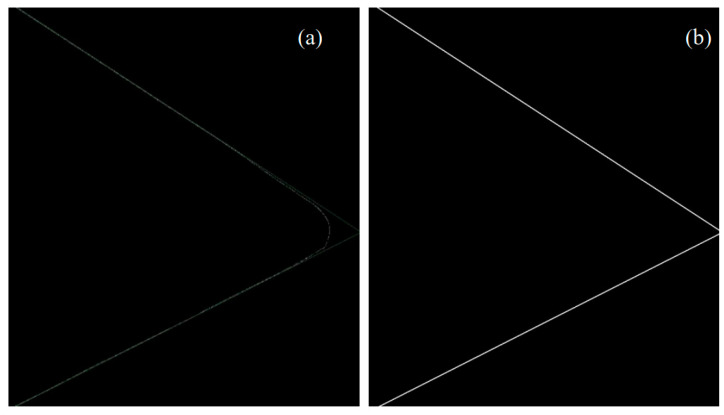
(**a**) Improved image of linear detection results. (**b**) Optimized rake face image of an external turning tool.

Detection results are optimized according to input features. For a rake face image, the input feature is the tool nose angle. LSD is carried out for the results of Canny edge detection as shown in Figure 3a. The detected straight lines of the main and secondary cutting edges on the tool nose are shown in green and extended. The white line represents the Canny pixel–level edge. The linear detection effect of the tool nose is better than only using LSD algorithm, and it is stronger to shield the interference of the tool shape. According to the slope of the two lines, the angle is calculated. Compared with the standard angle (60°) of the tool nose as the input feature, the angle error is obtained and further corrected.

Calculate the average error *ε* of the two lines that are the upper and lower Canny edges, respectively. When the angle is greater than 60°, rotate the line with a larger average error to a certain angle, which is conducive to reducing the angle between the two lines. Then calculate and compare the *ε* value again. Repeat the above process until the angle is 60°. When it is less than 60°, the line with a smaller *ε* is rotated by a certain angle, which serves to amplify the angle between the two lines. The value of *ε* is calculated and compared again. Repeat the above process until the angle is 60°. The image of the rake face is shown in Figure 3b, which is obtained by the optimized linear detection algorithm. 

Based on the improved line detection algorithm, the parametric equations are as follows, which relate the tool nose straight lines of the main and secondary cutting edges.

(8) $$k_{1}x+b_{1}y+c_{1}=0$$

(9) $$k_{2}x+b_{2}y+c_{2}=0$$

where $k_{1},{\;k}_{2},{\;b}_{1},{\;b}_{2},{\;c}_{1}$, and${\;c}_{2}$ are the parameters obtained by linear detection.

The distance is fixed between the tool nose center and the two straight lines, which is the standard radius *r* of the tool nose, and $r=\frac{\left|kx+by+c\right|}{\sqrt{k^{2}+b^{2}}}$. Bringing this equation into the linear equation of the main and the secondary cutting edges of tool nose, the following formula can be obtained:

(10) $$\left\{\begin{array}{c} \frac{\left|k_{1}x+b_{1}y+c_{1}\right|}{\sqrt{{k_{1}}^{2}+{b_{1}}^{2}}}=r\\ \frac{\left|k_{2}x+b_{2}y+c_{2}\right|}{\sqrt{{k_{2}}^{2}+{b_{2}}^{2}}}=r \end{array}\right.$$

**Figure 4:**
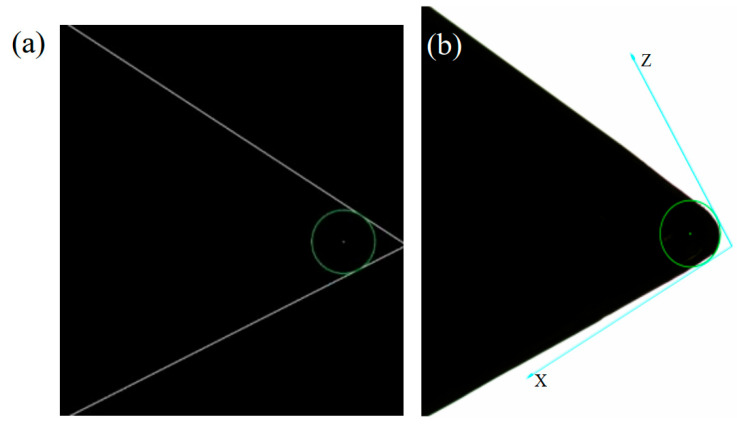
Detection results of tool nose theoretical arc. (**a**) Improved LSD algorithm to measure the circle center. (**b**) Drawing the theoretical arc on the original image.

The triangular external turning tool in this work is TNMG160404TSBB60, and its radius *r* is 0.4 mm. By solving Formula (10), four solutions for the possible position of the tool nose center can be obtained. The actual coordinate of the tool nose is located inside the tool profile, and then one correct solution is obtained, while the other three solutions are discarded. Figure 4 shows the green theoretical circle center, and draws a theoretical corner radius of 0.4 mm. The white line is the effect of improved algorithm detection (Figure 4a). The theoretical arc on the original image is given in Figure 4b.

**Figure 5:**
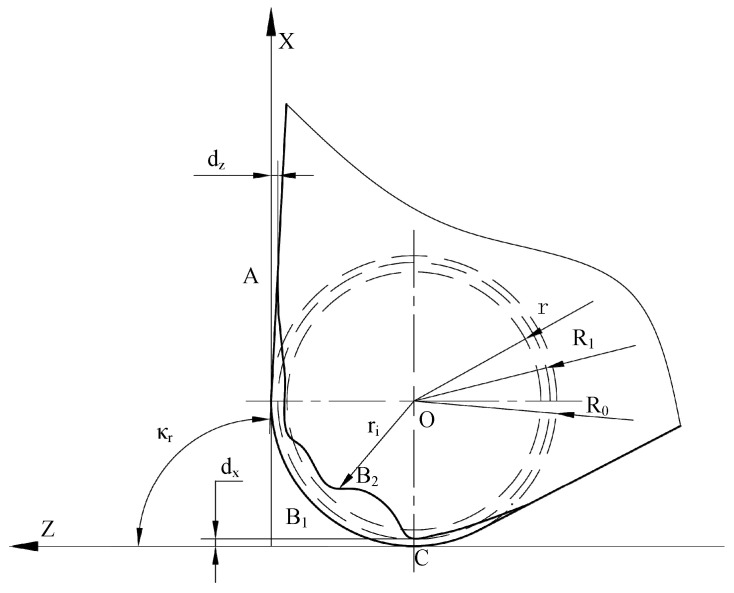
The actual angle of the tool nose geometry.

Figure 5 demonstrates the actual orientation of the geometric model after tool nose wear in a CNC machine. O is the center of the tool corner. *d_x_* and *d_z_* are the wear values in the X and Z directions, respectively. The arc AB_1_C is the shape of the tool nose without wear, and *R*_0_ is the corresponding standard radius. The curve AB_2_C is the tool nose shape after wear, and *R*_1_ is the corresponding theoretical radius after tool setting. *r*_i_ is the radius of point *i* on the tool corner after wear. *r* is the average radius after tool nose wear, and $r=\frac{\sum_{i=1}^{n}r_{i}}{n}$.

The distance *r*_i_ is calculated between the circle center and the tool nose contour located inside the theoretical corner. The average pixel radius after tool nose wear is obtained as 154.9826 pix. The pixel equivalent is *S* = 2.415 μm/pix, obtained by camera calibration, and the average pixel radius *r* is 0.3743 mm.

The average value of the minimum pixel distance is 5.5901 pix between the tool nose contour after wear and the *x-*axis or *z*-axis, which corresponds to the actual wear distance (0.0135 mm) eliminated by tool setting. The theoretical radius of the tool nose after tool setting is replaced by the unworn corner radius (0.4 mm) minus the wear distance, and then *R*_1_ = 0.3865 mm is calculated out. *R*_1_ and *r* will be employed for machining error compensation.

## 3. Tool Path Optimization and Machining Code Design for Corner Radius Error Compensation

### 3.1. Tool Path Optimization for the Error Compensation of Corner Radius

**Figure 6:**
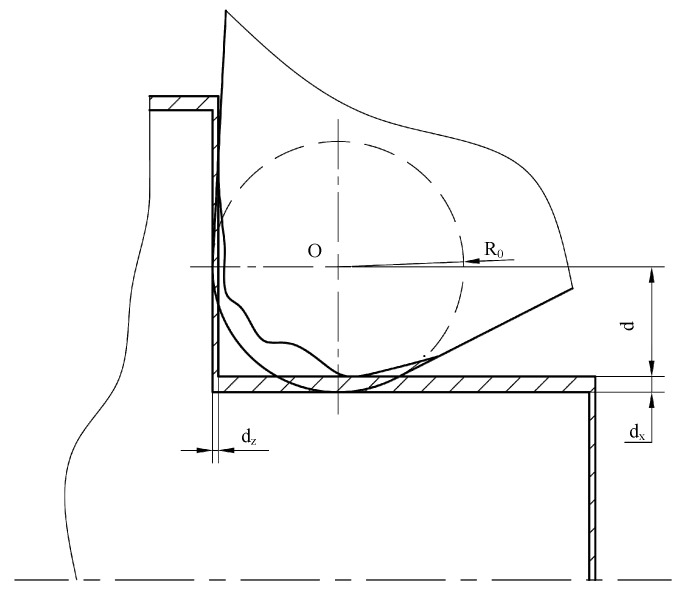
Batch processing error diagram.

The machining error caused by tool tip wear is reflected in the actual radius, which affects the size of the inner and outer cone and the arc surface. In batch machining with a single tool setting, tool wear will cause the axial length and the radial diameter to increase. *d*_x_ and *d*_z_ are the tool nose wear errors in the x-axis and z-axis directions, respectively (in Figure 6). It is necessary to ensure that the relative position of clamping workpieces is consistent each time. A strategy is adopted that the radial compensation is *d*_x_ in machining codes, and $d_{x}=R_{0}-d$, where *d* is as shown in Figure 6.

**Figure 7:**
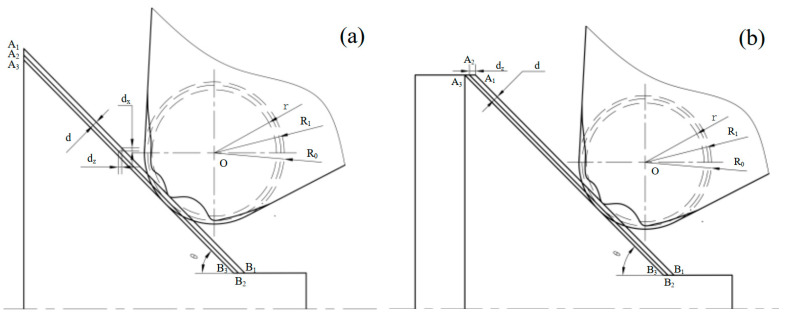
(**a**) The diameter error of the big end on the outer cone. (**b**) The front and back length error of the outer conical surface.

When machining the outer conical surface, turning tool wear will cause the maximum diameter of the conical surface to increase. In Figure 7a, A_3_B_3_ represents the machining conical surface trajectory by the theoretical corner arc. After tool nose wear, the cone surface moves outward to A_1_B_1_. In single workpiece machining, tool setting will eliminate the wear error in the X and Z direction, and the error is from A_3_B_3_ to A_2_B_2_. The error value is $d=R_{1}-r$, where *R*_1_ is the theoretical corner radius after tool setting, and *r* is the wear radius.

When the structure is as a cylinder behind the cone, the outer cone dimension remains unchanged, but the length of the cylinder behind the outer cone will increase *d*_z_ (in Figure 7b). The cylinder length in front of the outer cone will decrease *d*_z_, and the calculation equation is $d_{z}=\frac{d}{\sin\theta }$. Combining this with the equations mentioned above, we obtain $d_{z}=\frac{R_{1}-\frac{\sum_{i=1}^{n}r_{i}}{n}}{\sin\theta }$. The axial compensation *d*_z_ is taken to the machining codes, so that the cone A_1_B_1_ is translated back to A_2_B_2_. Horizontal cutting should be avoided when verifying the large conical diameter, but it is required when verifying the front and back length of the conical surface.

**Figure 8:**
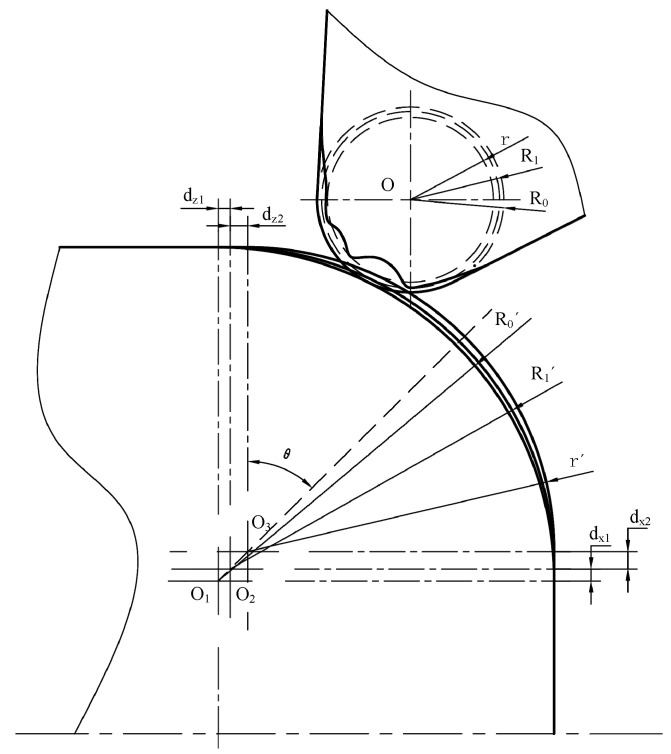
The schematic of convex arc radius error.

When machining a convex arc surface, tool wear will cause the arc radius to become smaller, the end surface diameter to be larger, and the cylinder length behind the arc to become longer. In Figure 8, $R_{0}^{\prime }$ is the arc surface radius of the theoretical tool nose machining. After tool nose wear, the arc surface shifts outward to *r’*. During single-workpiece processing, tool setting can eliminate the wear error from $R_{0}^{\prime }$ to $R_{1}^{\prime }$ in the X and Z directions and leave the error between $R_{1}^{\prime }$ and *r’*. The error of the arc surface radius is defined as *e*, and $e=R_{1}-r$. The error calculation formulas of the cylindrical length and end face, which are located in the front and back of the arc, are as follows:

(11) $$d_{x2}=e*\cos\theta$$

(12) $$d_{z2}=e*\sin\theta$$

where θ=45°. The radial compensation *d*_x2_ is implemented at the beginning point of the arc end face. The axial compensation is *d*_z2_ at the end point on the cylinder surface. Then the arc radius increases by *e*, so that it returns to the theoretical value *r’*.

### 3.2. CNC Machining Code Design

**Figure 9:**
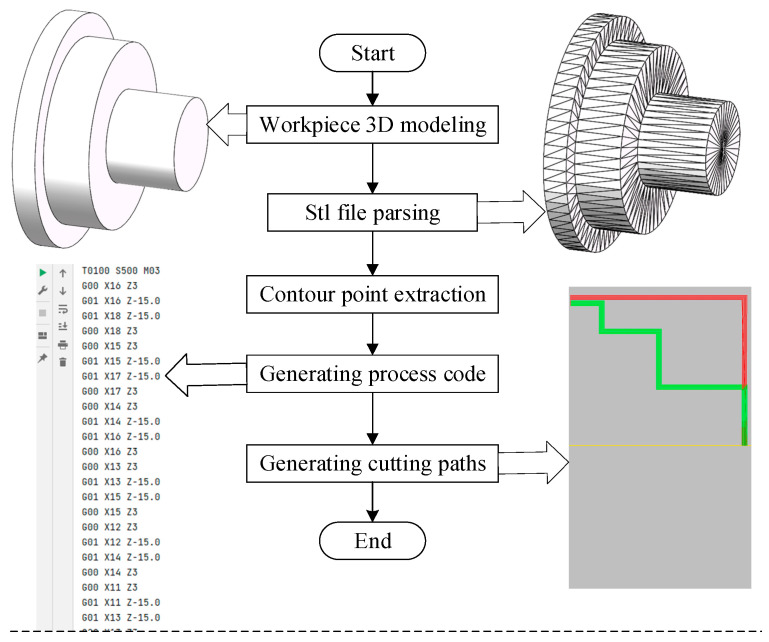
Process code generation flow. The green broken line in the lower right figure represents the cutting path of external turning tool.

The design and generation of CNC machining codes are mainly divided into three steps: STL 3D model analysis, rotary contour point extraction, and cutting path and machining code generation. (1) The third-party dependency library (numpy-stl) is used to complete the 3D model analysis of the STL (STereoLithography). The vertex coordinates and normal vectors of all triangular surfaces on the 3D model are obtained. (2) Determine the axis and direction of the rotating body according to the vertex coordinate of triangular surfaces and calculate the rotation radius of each contour point. (3) Generate cutting path and machining codes according to the rotary body profile. The overall process is displayed in Figure 9.

## 4. Error Compensation Experiments and Result Analysis

According to wear error compensation strategy of CNC turning machining, four kinds of workpieces are designed to complete experiments and verification. Workpiece 1 is used to verify the length compensation of the front and back cylinders in machining cone process. Workpiece 2 verifies the maximum diameter compensation, which should avoid horizontal cutting after machining the cone. Workpiece 3 proves the diameter compensation in machining arc surfaces. Workpiece 4 is utilized to verify cylindrical diameter compensation during batch machining with the strategy of single tool setting.

**Figure 10:**
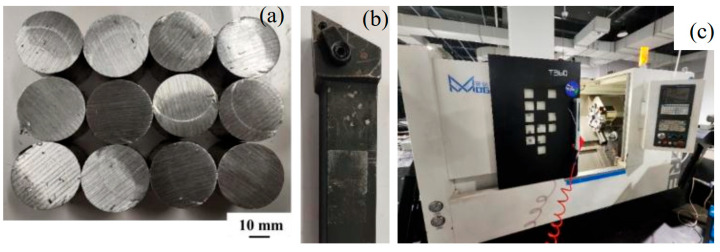
(**a**) Some workblanks. (**b**) External turning tool. (**c**) T360 CNC horizontal lathe.

The processed material used in experiments is 6061-T6 aluminum alloy with a diameter of 32 mm. Some workblanks are shown in Figure 10a. The main declination *κ*_r_ of the external turning tool is 93°. The tool is demonstrated in Figure 10b, and it is made of ceramic material. The machine tool is a T360 CNC horizontal lathe (Figure 10c). Its controller model is GSK-980TDi-plus, produced by GSK CNC Equipment Co. Ltd in Guangzhou, China. The technical parameters and machining accuracy are exhibited in Table 2 and Table 3, respectively. The error compensation processing is provided with the cooling effect by the cutting fluid system of the CNC machine.

**Figure 11:**
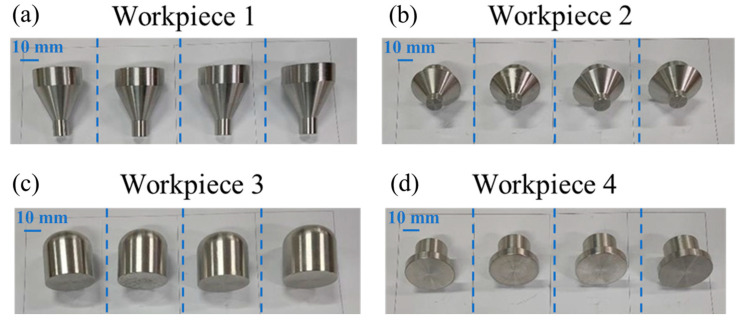
Experimental workpieces before compensation. (**a**) Workpiece 1. (**b**) Workpiece 2. (**c**) Workpiece 3. (**d**) Workpiece 4.

For workpiece 1, the measured corner wear radius is 0.3743 mm, and the theoretical radius after tool setting is 0.3865 mm. The calculated axial compensation is 0.03 mm. The arc surface radius is measured with a digital display radius gauge (accuracy, 0.01 mm). Four workpieces 1 specimens were processed using machining codes before compensation, as shown in Figure 11a. The cylindrical surface length behind the large conical surface is 10 mm, measured by a micrometer, and the calculated error is shown in Table 4. The average length error before compensation is 0.029 mm, and it is 0.007 mm after compensation.

Due to its different cone angle from that of workpiece 1, the recalculated axial compensation of workpiece 2 is 0.02 mm. Four workpieces were processed using the machining codes before compensation (Figure 11b). We measured the maximum diameter of the large conical surface and calculated the error. The results are shown in Table 4. The average diameter error of the big cone before compensation is 0.024 mm, and it is 0.005 mm after compensation.

For workpiece 3 (Figure 11c), the calculated radial compensation *d*_x2_ is 0.011mm, the axial compensation *d*_z2_ is 0.011 mm, and the incremental value of the arc radius *e* is 0.015 mm. Four workpieces were machined separately using the processing codes before and after compensation. Each measurement was rotated on the circular surface. The error is calculated as shown in Table 4. The average diameter error of the large cone before compensation is 0.02 mm, and it is 0.013 mm after compensation.

Due to the point contact between the circumference of the large conical surface and the measured surface, there is a small measurement error in experimental workpieces. To verify the error compensation more accurately, workpiece 4 was designed (Figure 11d), and its cylindrical diameter was measured. Workpiece 4 worked under single tool setting for batch machining to improve efficiency.

The specific method is that a new tool is utilized to set and simulate batch processing, then the tool nose wear value is measured. Workpiece 4 was facing the machining tool to eliminate the axial error. The three-jaw chuck ensures that the radial position of each clamping is unchanged. Then the radial wear value *d*_x_ of 0.016 mm was obtained. The processing codes before and after radial compensation *d*_x_ were used to fabricate four workpieces separately. Furthermore, the cylindrical diameter (30 mm) of the step shaft was measured. Then error value was calculated. The results are shown in Table 4. The average cylindrical diameter (ø30 mm) error before compensation is 0.017 mm, and it is 0.005 mm after compensation.

The summary of average error reduction rates for various cases, from workpiece 1 to workpiece 4, is also shown in Table 4. In general, the processing error has been significantly reduced (maximum 79.2%). In contrast, the error compensation model of CNC was established through the traditional homogeneous coordinate transformation matrix (HTM) in 2023, which contained two-dimensional angle errors. Experiment results showed that the body-diagonal accuracy after compensation was 54.82%, which was higher than that of the traditional model [28]. Position-dependent geometric errors were derived using error identification equations based on polynomials. A regularization method was then selected to identify geometric error parameters. Experiment results revealed that the final volumetric diagonal positioning error with compensation was controlled at 20 μm (approximately 46%) [29].

Compared with other studies in similar fields, this research has achieved better machining error compensation effects for CNC external turning tool.

## 5. Web Display Platform of Wear Error Compensation System

The operating system of the experiments was Windows 11, the development language was Python (3.9), and the programming software was PyCharm (2021.2.2). To effectively manage Python and third-party libraries, the Anaconda tool was chosen. Furthermore, based on front-end SpringBoot and back-end ReactJS development framework, a powerful Web platform was developed, which mainly included four interfaces of product information, tool information, intelligent compensation, and CNC management. It can realize the visual display of processing codes and tool paths.

The product information interface is mainly used to define new machining parts, which require uploading the workpiece model and the process card. The tool information interface manages various machining tools, including addition and removal tools. The intelligent compensation interface will select the machining tool for the workpiece, and the CNC can be selected at the same time. The CNC management page is utilized to define new machines.

The tool wear condition was monitored during the machining gap, and the data was uploaded through the customized cloud platform. Then tool images were preprocessed, and the wear types were analyzed. Pixel-level image annotation tool of Labelme marked the wear area of the flank face. With the help of Pytorch and other third-party libraries, the deep learning model was constructed and trained, which helped realize accurate extraction of wear regions and automatic classification of tool wear states. Then tool wear value was obtained, and the error compensation of tool nose wear was achieved.

**Figure 12:**
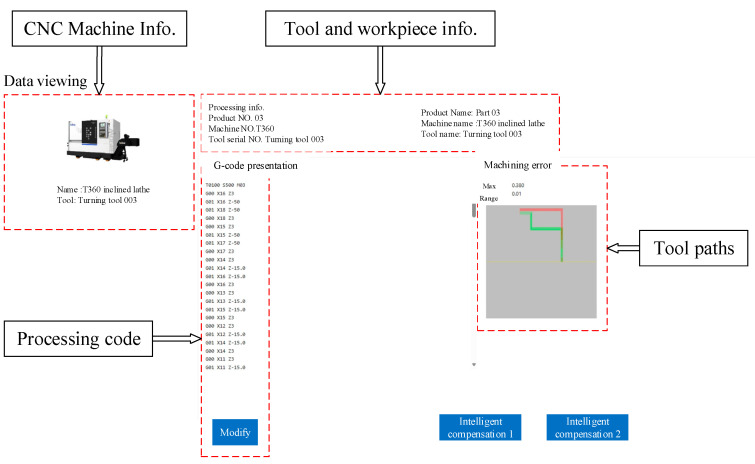
Intelligent compensation processing codes and tool path generation interface.

The intelligent compensation function module is divided into intelligent compensation 1 and compensation 2. Intelligent compensation 1 is implemented in workpieces containing a cone. The interface will display the workpiece 3D model, processing information, CNC information, etc. Clicking the processing result button will generate the uncompensated codes. Clicking the intelligent compensation 1 button will generate the machining codes after compensation, with the machining tool path displayed on the left side of the page. The compensation effect is verified by machining workpiece 1 and workpiece 2. Intelligent compensation 2 is utilized for single tool setting in batch processing. The generating page demonstrates processing codes and cutting paths, as shown in Figure 12. The compensation effect is proved by machining workpiece 4.

## 6. Conclusions

A post-treatment process is proposed for the wear area of the main flank face in this work. The wear value was measured, and tool wear states were judged according to the blunting standard. For an external turning tool that has not reached the wear standard, a detecting straight-line method of the main and secondary cutting edges is proposed based on edge images and input features. The theoretical center of the tool nose and morphological visual modeling were completed. Then actual wear radius was calculated. The influence of tool nose wear on machining accuracy was analyzed. The axial and radial compensation of different wear conditions were carried out by establishing a variety of mathematical models.

Some kinds of machining workpieces were designed, and experiments were conducted to verify the effectiveness of different compensation strategies. By comparing the processing data before and after compensation in experiments, intelligent compensation was proven to reduce machining errors. Compensation strategies have reduced the average errors of various workpiece types by 75.8%, 79.2%, 37.5%, and 69.6% and significantly improved the machining accuracy. An intelligent compensation management system was further developed based on a Web platform. The system provides comprehensive processing management and displays functions through the modules of product information, tool information, intelligent compensation, and CNC management.

For further studies, intelligent compensation algorithms will continue to be improved. Considering more parameters, such as cutting speed and depth and workpiece materials, is beneficial for enlarging the applicability of the compensation method and achieving more accurate compensation effects.

## Figures and Tables

**Table 1:** Flank face wear value of a CNC external turning tool.

	Wear Type	Slight Wear (mm)	Uniform Wear (mm)	Non-Uniform Wear (mm)
Wear Index				
*VB*		0.048	0.156	0.190
*VB*_max_		0.079	0.241	0.429

**Table 2:** Technical parameters of T360 CNC horizontal lathe.

Items	Specification
Maximum rotary diameter	560 mm
Maximum cutting length	500 mm
Maximum cutting diameter	360 mm
Main shaft hole diameter	65 mm
Maximum machining bar diameter	50 mm
*X*-axis stroke	200 mm
*Z*-axis stroke	560 mm
Tailstock stroke	450 mm
External turning tool size	25 × 25 mm
Mainshaft speed range	50~4500 r/min

**Table 3:** Machining accuracy.

Parameters	Specification (mm)
Positioning accuracy (*X*-axis)	0.008
Positioning accuracy (*Z*-axis)	0.008
Repeated positioning accuracy (*X*-axis)	0.004
Repeated positioning accuracy (*Z*-axis)	0.005

**Table 4:** The micro-error before and after compensation of different experimental workpieces.

Workpiece Type		Error Type	Error Value (mm)				Average Error Reduction Rate
1	Length error compensation of cone	Original	0.034	0.036	0.020	0.026	75.8%
		Compensated	0.005	0.009	0.008	0.007	
2	Diameter error compensation of large conical surface	Original	0.022	0.026	0.028	0.019	79.2%
		Compensated	0.005	0.004	0.007	0.002	
3	Radius error compensation of the arc surface	Original	0.02	0.01	0.02	0.03	37.5%
		Compensated	0.01	0.01	0.01	0.02	
4	Diameter error compensation of cylindrical surface	Original	0.014	0.020	0.016	0.019	69.6%
		Compensated	0.006	0.008	0.005	0.002	

## Data Availability

The raw data supporting the conclusions of this article will be made available by the authors on request.
